# Opsoclonus-Myoclonus Syndrome in Children and Adolescents: A Therapeutic Challenge

**DOI:** 10.3390/children8110965

**Published:** 2021-10-26

**Authors:** Marina Auconi, Laura Papetti, Claudia Ruscitto, Michela Ada Noris Ferilli, Fabiana Ursitti, Giorgia Sforza, Federico Vigevano, Massimiliano Valeriani

**Affiliations:** 1 Child Neurology and Psychiatry Unit, Systems Medicine Department, Tor Vergata University of Rome, 00133 Rome, Italy; marina.auconi@opbg.net (M.A.); claudia.ruscitto@opbg.net (C.R.); 2 Child Neurology Unit, Neuroscience Department, Bambino Gesù Children’s Hospital, IRCCS, 00165 Rome, Italy; laura.papetti@opbg.net (L.P.); michela.ferilli@opbg.net (M.A.N.F.); fabiana.ursitti@opbg.net (F.U.); giorgia.sforza@opbg.net (G.S.); federico.vigevano@opbg.net (F.V.); 3 Center for Sensory-Motor Interaction, Aalborg University, 9100 Aalborg, Denmark

**Keywords:** opsoclonus-myoclonus syndrome, pediatric neuroimmunological disorder, neuroblastic tumors, treatment, outcome, children

## Abstract

Opsoclonus-myoclonus syndrome (OMS) is a neurological non-fatal disease that usually responds to immunotherapies. However, the real challenge is to counteract the high frequency of relapses and long-term developmental sequelae. Since the OMS is extremely rare, a common consensus regarding therapeutic guidelines is still lacking. The goals of this study were to test whether ACTH was superior to other immunotherapies and to investigate whether an early treatment could improve the outcome. Sixteen children affected by OMS were retrospectively reviewed. Eight children had a neuroblastic tumor. The other eight patients were affected by non-paraneoplastic OMS. Overall, the most commonly used treatment was corticotherapy (*n* = 11). However, ACTH (*n* = 10), rituximab (*n* = 7), immunoglobulins (*n* = 4), cyclophosphamide (*n* = 3), and mycophenolate (*n* = 2) were also administered. ACTH was associated with a high percentage of patients who healed (80%) and, as a first-line therapy, was associated with a lower incidence of relapses. An early treatment was associated with a favorable long-term outcome. Long-term sequelae occurred in 42% of patients who were treated early and in all of those who were treated late. It is advisable for the affected children to be identified at an early time, as they may benefit from an early treatment. ACTH represents an effective treatment with a high probability of recovery and low rate of relapses.

## 1. Introduction

Opsoclonus-myoclonus syndrome (OMS), also known as “dancing eye syndrome”, is a rare neurological disorder characterized by rapid, involuntary, chaotic eye movement (opsoclonus), myoclonus, and ataxia. Opsoclonus consists of sudden, involuntary, chaotic, arrhythmic, and multidirectional (upwards, downwards, and torsional) conjugate saccadic ocular movements. OMS is often associated with additional manifestations, such as irritability and sleep disturbances.

The etiology is still not clear and is different between children and adults. In about 50% of pediatric cases, the disorder is associated with neuroblastoma or ganglioneuroblastoma, with abdominal or thoracic localization, and generally with favorable histological features [1]. The symptoms are thought to develop as a result of the autoimmune response triggered by the tumor [2]. In the remaining 50% of cases, OMS develops secondarily to a viral infection or to an immune response triggered by an unknown agent [3]. Some authors formerly proposed that idiopathic OMS can be triggered by an already regressed neuroblastoma, even if it is no longer detectable [4,5]. In adults, the percentage of OMS triggered by cancer is higher (60%). The tumors most frequently involved are small cell lung carcinoma, breast, and ovarian cancers [6].

The incidence of pediatric OMS is 0.18 cases per million total population per year [7]. The mean age at presentation is 18 months (range of 3–42 months) [7].

The lack of specific criteria for the identification of OMS disease leads to a delay in diagnosis. Due to the earlier appearance of ataxia, OMS is often misdiagnosed as acute cerebellar ataxia, especially when opsoclonus is not detectable. The differentiation of OMS from acute cerebellar ataxia is crucial since, according to some studies, early diagnosis and treatment may prevent some residual neurological and behavioral consequences of this disease [8,9,10,11].

Since the OMS is extremely rare, no randomized control trials concerning the treatment are available and, accordingly, therapeutic guidelines are missing. At the present time, immunotherapies such as steroids, adrenocorticotropic hormone (ACTH), intravenous immunoglobulin (IVIG), and different other therapies (cyclophosphamide, plasmapheresis, rituximab) are the main tools for the treatment of neurological symptoms [12,13]. Furthermore, despite therapy, 70% of children may have clinical relapses [14], often due to intercurrent illness [3] or treatment tapering [15].

Remarkably, the outcome seems to be independent of the presence of neuroblastoma.

In the present retrospective study, we describe the clinical characteristics and outcome of 16 pediatric patients with OMS. We aimed to investigate whether an early treatment could improve the outcome and to test whether ACTH was superior to the other immunosuppressant drugs.

## 2. Materials and Methods

### 2.1. Patients

Medical charts of 16 children (4 males and 12 females) with OMS, consecutively hospitalized in Bambino Gesù Childrens’ Hospital from January 2007 to December 2020, were reviewed. The mean age of the patients was 32.2 months (range of 8–186 months). Their clinical and demographic characteristics are shown in Table 1. Age at onset, sex, neurological symptoms, diagnosis, treatments, and outcomes were considered. Moreover, the interval between the onset of the symptoms and the start of treatment was assessed. The diagnosis was made by paediatric neurologists from our department, based on the criteria defined at the Genova meeting in 2004 [16], namely the presence of at least three of the following four features: (1) Opsoclonus, (2) sudden, brief, shock-like muscle spasms (myoclonus) or ataxia, (3) behavioral change and/or sleep disorders, and (4) neuroblastoma. Persistence, after 1 year of treatment, of motor impairments, such as opsoclonus, ataxia, and/or myoclonus, was considered as a neurological sequela. Neuropsychiatric sequelae were defined as the persistence of symptoms, such as language disturbance, cognitive delay, and behavioral disorders at 1 year after the treatment.

Moreover, we took into account the putative trigger of OMS (tumors, viral infection, etc.). The diagnosis of the tumor was confirmed by a histopathological examination. The tumor location was abdominal or thoracic (Table 1). The tumor was in the pelvic area in only in one case. In particular, the tumors included one pelvic neuroblastoma (case 1), two ganglioneuroblastomas in the left paravertebral subrenal area (cases 2 and 6), left adrenal retroperitoneal neuroblastoma (cases 3 and 8), left adrenal stroma-poor neuroblastoma (case 4), right parailiac neuroblastoma (case 5), and schwannian stroma-poor neuroblastoma of the posterior mediastinum in the left paravertebral location (case 7).

### 2.2. Statistics

The prevalence of different categories for each variable was analyzed. Chi-square (ꭓ²) statistics were used to determine the difference in the distribution of categorical variables among the study groups. Z-score statistics were used to determine the difference in the distribution of numerical variables. A *p*-value of <0.05 was considered statistically significant.

## 3. Results

### 3.1. Radiological and Neurophysiological Examinations

All of the patients underwent brain MRI or TC, which resulted as normal. In addition, all of the patients underwent chest-abdomen CT or MRI. In eight children, neuroblastoma or ganglioneuroblastoma were found. The other eight patients were affected by non-paraneoplastic OMS. All of the patients were tested for the presence of neurotropic virus Herpes simplex, Varicella-Zoster, Epstein-Barr, Cytomegalovirus, HHV-6, Parvovirus, Coxsackie, Adenovirus, and auto-antibodies in cerebrospinal fluid (CSF). Two children were found positive for HHV-6 and two patients were diagnosed with anti-N-methyl-D-aspartate receptor (NMDAr) encephalitis, while for the other four patients the trigger factor remained unknown. Nine of the sixteen patients underwent electroencephalography (EEG) that resulted as normal in seven subjects. In a child affected by neuroblastoma, EEG was characterized by slow waves in the right temporal region, while in the remaining patient who is affected by the anti-N-methyl-D-aspartate receptor (NMDAr) antibody encephalitis, EEG showed an asymmetric cerebral electric activity with slow waves on the left hemisphere.

### 3.2. Clinical Characteristics

The clinical characteristics of OMS patients are shown in Table 1. In our cohort, the most frequent symptoms were myoclonus (100% of cases) and ataxia (94%). Opsoclonus was present in 75% of patients (12/16). In several cases, opsoclonus occurred at a later time, which partly explains the delay in diagnosis. Behavioral disorders were common (75% of cases) and three patients had sleeplessness. No differences were detectable in the incidence of symptoms between the paraneoplastic versus non-paraneoplastic OMS group (*p* = 0.3 for ataxia, *p* = 0.25 for opsoclonus, *p* = 0.25 for behavioral disorders, and *p* = 0.13 for insomnia).

### 3.3. Treatment and Outcome

The treatment differed between patients with paraneoplastic OMS and patients with idiopathic OMS.

Eight patients affected by the tumor were treated with a multimodal approach, based on the tumor removal and followed by a 12-month-cycle of corticosteroids. Specifically, they received dexamethasone (20 mg/m^2^/die, 3 consecutive days per month for 12 months). Three patients (37.5%) had a good response, while the remaining five (62.5%) had a neurological relapse and required a second-line medication. In the latter group, two patients recovered after the ACTH and cyclophosphamide treatment, respectively. The remaining three children are still under treatment, with rituximab, chemotherapy (etoposide and carboplatin), as well as ACTH and steroids, respectively. They did not achieve full recovery from the neurologic symptoms.

Among the patients with non-paraneoplastic OMS, three were treated with ACTH with an excellent response and remission of neurological symptoms.

The ACTH scheme consisted of daily intramuscular administration at a dosage of 0.1–0.2 mg/day for 2 weeks followed by a dose every 2 days for 1 month. Then, a dose every 3 days for 2 months and thereafter, according to the clinical trend. The choice of the daily dosage depended on the weight of the child: Under 10 kilos of weight, the dose was 0.1 mg/day, while over 10 kilos of weight, the dose was 0.2 mg/day.

One of these patients subsequently undertook therapy with rituximab and mycophenolate, due to the diagnosis of anti-NMDAr encephalitis, although the neurological symptoms had not relapsed. One patient was initially treated with a cycle of intravenous Ig in monotherapy, but the positive effects lasted only 15 days after every cycle. The patient was subsequently treated with ACTH and obtained a remission of symptoms. Four patients underwent corticosteroids and obtained a poor response. Two of them recovered after the ACTH treatment, while the others recovered after the treatment with rituximab.

**Figure 1:**
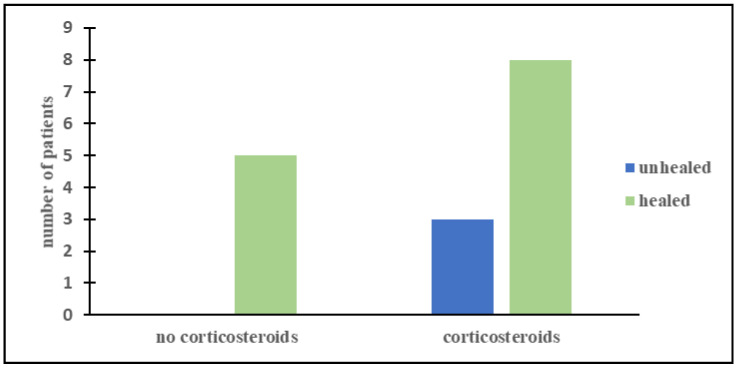
Comparison between the outcomes of patients who either did not use (**left columns**) or used (**right columns**) corticosteroids.

**Figure 2:**
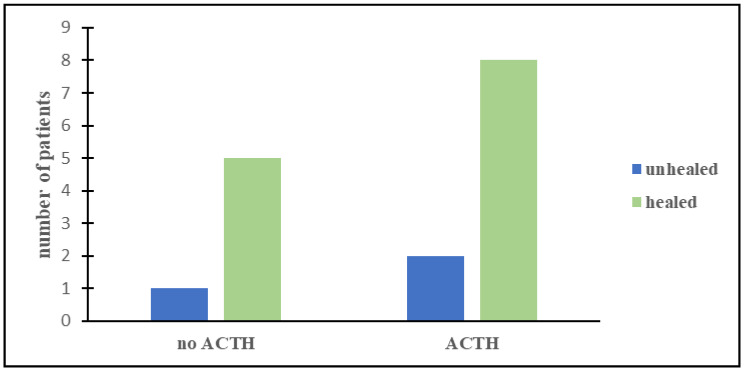
Comparison between the outcomes of patients who either did not use (**left columns**) or used (**right columns**) ACTH.

Most of our patients were treated with more than one immunotherapy (Table 2 and Table 3). Corticosteroids were the most frequently administered drug (68.8% of patients) and proved to be very effective in 8/11 patients (73%). However, we did not find a statistically significant advantage of corticosteroids over the other immunotherapies (*p* = 0.2, Figure 1). Three patients who took ACTH as a first-line treatment had a prompt remission of neurologic symptoms, no relapses, and an excellent outcome. Moreover, five children who had not responded to the high-dose oral corticosteroids improved after a second-line therapy with ACTH. In total, ACTH was used in 10 patients and a long-term resolution of all the neurological symptoms was achieved in 80% of the cases (Figure 2). However, the advantage of ACTH over the other immunosuppressors could not be confirmed statistically (*p* = 0.9), probably due to the small number of patients. Other immunotherapies (IVIG, rituximab, cyclophosphamide, and mycophenolate) were more rarely administered. Therefore, reliable information on their effectiveness cannot be issued from our population. In our group, more than half of the children had neurological relapses after the first treatment (10/16) (63%). Interestingly, no patient who had ACTH as a first-line treatment showed a relapse. A statistically significant correlation was observed between the use of ACTH as a first-line treatment and the lower incidence of relapses (*p* = 0.01).

No significant side effects were observed in patients treated with corticosteroids or immunosuppressants. Only one patient treated with ACTH showed mild irritability, while two patients had weight gain during the treatment period. The side effects subsided after the withdrawal of ACTH.

Among our patients, 13 have had a complete recovery of neurological capabilities and are currently neurologically normal. The remaining three patients had long-term neurological sequelae (cases 4 and 6 still show ataxia and case 7 shows tremor and ataxia) (Table 1). Seven children showed neuropsychiatric sequelae, including expressive language disorders (31%), cognitive disabilities (19%), and irritability (12,5%). One patient developed a severe anxious-depressive symptomatology that required hospitalization in the Psychiatric department of our hospital. Overall, nine patients had long-term neurological and/or neuropsychiatric sequelae, while seven patients had a favorable outcome with no sequelae at all.

We tested the hypothesis that an early treatment could avoid the persistence of neurological or neuropsychiatric sequelae. In our patients, the mean time that elapsed between the onset of the symptoms and the start of treatment was 33.44 days (ranging from 1 to 240 days). The treatments that started before and after 15 days from the disease onset were considered as “early” and “late”, respectively. Therefore, 12 patients were early-treated and four patients were late-treated. Among the twelve patients treated early, 42% had neurological and/or neuropsychiatric sequelae (five patients) and 58% did not (seven patients). Neuropsychiatric sequelae included mild cognitive disabilities. All of the four late-treated patients developed sequelae (100%). The mean interval time between the OMS onset and treatment of patients with sequelae was 50.7 (±80) days, while it was 11.3 (±5.4) days for the patients who have fully recovered. The difference approached the statistical significance (*p* = 0.07).

## 4. Discussion

OMS is a non-fatal disease that usually responds to immunotherapies. However, the real challenge is to counteract the high frequency of recurrence and sequelae. The most effective drug has not been defined yet and a common consensus regarding therapeutic guidelines is still lacking. This is mainly due to the poor knowledge of the pathophysiologic mechanisms of the disease. The pathogenesis of OMS involves the activation of the immune system and the elevation of T and B cells and other neuroinflammatory elements in CSF and blood [17]. This observation has led to the use of corticosteroids as a first line of therapy, even for a prolonged time. However, the corticosteroids treatment is associated with numerous OMS relapses and long-term sequelae [18] and, if prolonged, can lead to severe side effects. Studies [12,13] have reported that a multimodal treatment, including corticosteroids, is to be considered as a first-choice therapy. In a group of seven patients with paraneoplastic OMS, Mizia-Malarz et al. [12] showed that the combined treatment with corticosteroids and cyclophosphamide resulted in a complete resolution of OMS symptoms in six children. The authors showed that in patients with neuroblastoma, tumor resection is not sufficient per se to resolve the symptoms, but it must be supported by a multimodal pharmacological treatment. In a retrospective study of 19 patients, Pranzatelli and Tate [13] showed that a combined immunotherapy based on dexamethasone, IVIG, and rituximab reduced the neuroinflammation caused by OMS, leading to a significant reduction of B cells in CSF (reduction of 95%) and blood (75%). The comparison group, treated with dexamethasone alone or dexamethasone and IVIG, showed a partial clinical response and the presence of several neuroinflammatory markers (including expansion of CSF B cells, increased concentrations of CSF CXCL13 and CXCL10, and serum CCL22), despite a mean therapy duration of 7 months.

Although the role of ACTH in the OMS treatment has been well established since 1962 [19], very few studies have investigated its efficacy. An early study by Koh et al. reported that the ACTH monotherapy was associated with a 90% relapse rate [1]. The authors concluded that a polytherapy involving several immunosuppressive drugs could be more effective than ACTH alone. Tate et al. [20] carried out a large controlled study to compare the efficacies of ACTH-based immunotherapies in a large group of children with OMS. They demonstrated a greater efficacy of ACTH than the corticosteroid-based therapy and a greater response of ACTH-based multimodal therapy (ACTH combined with rituximab, chemotherapy or a steroid sparer) compared with ACTH alone or with IVIG.

ACTH could work through a multiplicity of mechanisms, some immunological, others neural. Recently, corticotropin was found to reduce the elevated levels of B cell activating factor in opsoclonus-myoclonus cerebrospinal fluid [20].

A study by Hammer et al. showed that a therapeutic response can be obtained only with ACTH, both for initial symptoms and relapses [21]. In our sample, we found a high percentage of patients who healed (80%) among those treated with ACTH. The two patients that did not heal were affected by paraneoplastic OMS. Furthermore, the use of ACTH as a first-line therapy was associated with a lower incidence of relapses.

A special note should be reserved for rituximab. Rituximab is a monoclonal antibody against CD20, which is expressed on B cells. Based on the identification of B cell expansion in many OMS patients by Pranzatelli et al. [17], rituximab may represent an effective and promising drug. Two studies [22,23] showed excellent results in OMS patients, especially in cases with severe symptoms and a relapsing course. In our cohort, rituximab has been used as a second-line treatment in six patients with good results.

Despite the proven role of T cells in the OMS pathogenesis, suggested by the expansion of Gamma delta T cells subsets and the lower percentage of CD4+ T cells in CSF [17], targeted therapies that alter the T cell quantity or function, such as mycophenolate mofetil, are poorly studied in the context of OMS.

Some previous studies suggested that a prompt treatment is associated with a better outcome. De Grandis et al. reported a higher frequency of neurological sequelae in children treated after 2 months from the symptom onset than in those treated at an earlier time [9]. Similarly, Hasegawa et al. found less severe neurological sequelae in patients treated within 30 weeks than in those treated at a later time [8]. A recent study in patients affected by neuroblastoma suggested that the early detection and treatment of tumor might improve the neurological outcomes [10]. Conversely, other studies suggest that a negative outcome is not significantly prevented by an early treatment [1,11,14]. Koh et al. [1] did not find any apparent relation between the duration of neurological symptoms before the diagnosis and symptom outcome. Mitchell et al. [14] suggested that developmental sequelae are not adequately prevented by the earlier and intensive treatment. According to Pohl et al. [11], the outcome is independent of a delay in the treatment. Our data showed that among the patients who were treated at an early time (12 patients), 42% of them developed long-term neurological and/or neuropsychiatric sequelae (six patients), which were present in all of the patients who were treated at a later time. Therefore, our data support the possible positive influence of an early treatment on the OMS outcome.

## 5. Conclusions

The present study shows that an early treatment is associated with a better prognosis with the reduced risk of neurological or neuropsychiatric sequelae. Regarding the type of drug, the patients who were treated with ACTH compared to corticosteroids showed a lower frequency of relapses and sequelae than those treated with other corticosteroids or an immunosuppressant. Although the response rate to the ACTH therapy for OMS is high, the general neurological prognosis is poor for more than half of the patients. Finally, it is not possible to draw unambiguous recommendations since it is a retrospective review with a few described patients. Furthermore, the generalization of the data is not possible. Therefore, additional studies are worthy of consideration.

## Figures and Tables

**Table 1:** Demographic and clinical characteristics of patients with opsoclonus-myoclonus syndrome.

Patients	Gender	OMS Etiology	Age at OMS Onset	Interval between OMS Onset and Initial Therapy (Days)	Interval between Therapy and OMS Remission (Months)	Neurological and Neuropsychiatric Outcomes	OMS Symptoms at Present
CASE 1	F	Pelvic Neuroblastoma	8 mo	15	4	Normal	Disappeared
CASE 2	F	Left paravertebral Ganglioneuroblastoma	1 y 10 mo	240	12	Speech delay	Disappeared
CASE 3	F	Left adrenal Neuroblastoma	1 y 8 mo	1	52	Normal	Disappeared
CASE 4	F	Left adrenal Neuroblastoma	2 y 5 mo	3	Remission never achieved	Speech delay; mild ataxia; postural instability	Residual
CASE 5	F	Right parailiac Neuroblastoma	1 y 10 mo	120	7	Behavioral disorder	Disappeared
CASE 6	M	Left paravertebral Ganglioneuroblastoma	2 y	1	Remission never achieved	Mild ataxia	Residual
CASE 7	F	Left paravertebral Neuroblastoma	1 y 8 mo	15	Remission never achieved	Mild tremor; mild ataxia	Residual
CASE 8	M	Left adrenal Neuroblastoma	1 y 5 mo	15	1	Normal	Disappeared
CASE 9	F	Unknown etiology	1 y 3 mo	31	45	Specific learning disability; mild cognitive delay	Disappeared
CASE 10	M	Unknown etiology	4 y	1	40	Language impairment; emotional dysregulation	Disappeared
CASE 11	F	Probable post-infectious aetiology (HHV6)	1 y 11 mo	10	7	Normal	Disappeared
CASE 12	M	Probable post-infectious aetiology (HHV6)	1 y 6 mo	30	4	Language impairment; psychomotor delay	Disappeared
CASE 13	F	Unknown etiology	1 y 5 mo	15	37	Normal	Disappeared
CASE 14	F	Unknown etiology	1 y 6 mo	8	1	Normal	Disappeared
CASE 15	F	NMDAr	15 y 6 mo	15	2	Anxious-depressive symptomatology	Disappeared
CASE 16	F	NMDAr	2 y 4 mo	15	2	Normal	Disappeared

Abbreviations: OMS: Opsoclonus-myoclonus syndrome; HHV-6: Human herpesvirus-6; NMDAr: Anti-N-methyl-D-aspartate receptor; mo: Months; y: Years.

**Table 2:** Treatments administered in paraneoplastic (cases 1–8) and non-paraneoplastic (cases 9–16) patients. Abbreviations: ACTH: Adrenocorticotropic hormone; IVIG: Intravenous immunoglobulin; RX: Rituximab; CP: Cyclophosphamide; MMF: Mycophenolate mofetil. “+” means that the treatment was administered; “-“ means that the treatment was not administered.

Patients	Tumor Removal	Steroids	ACTH	Rituximab	IVIG	CP	MMF
CASE 1	+	+	-	+	-	-	-
CASE 2	+	+	-	-	-	-	-
CASE 3	+	+	+	-	-	-	-
CASE 4	+	+	-	-	-	-	-
CASE 5	+	+	-	-	-	-	-
CASE 6	+	+	+	+	+	+	-
CASE 7	+	+	+	-	-	-	-
CASE 8	+	+	-	-	-	+	-
CASE 9	-	-	+	-	+	-	-
CASE 10	-	+	+	+	+	+	-
CASE 11	-	+	+	-	-	-	-
CASE 12	-	+	+	+	-	-	-
CASE 13	-	-	+	-	-	-	-
CASE 14	-	-	+	-	-	-	-
CASE 15	-	-	-	+	+	-	+
CASE 16	-	-	+	+	-	-	+

**Table 3:** The percentage of patients that had undergone treatment with the different immunosuppressor drugs.

Treatment	No. of Treated/16 Patients	%
Corticosteroids	11	68.8
ACTH	10	62.5
Rituximab	7	37.5
IVIG	4	25
Cyclophosphamide	3	18.8
Mycophenolate mofetil	2	12.5

## Data Availability

Data used to support the findings of this study are available from the corresponding author upon request.
